# Conducting Community Audits to Evaluate Community Resources for Healthful Lifestyle Behaviors: An Illustration From Rural Eastern North Carolina

**Published:** 2011-10-15

**Authors:** Jared T. McGuirt, Stephanie B. Jilcott, Maihan B. Vu, Thomas C. Keyserling

**Affiliations:** Center for Health Promotion and Disease Prevention, University of North Carolina at Chapel Hill. Mr McGuirt was at East Carolina University, Greenville, North Carolina, at the time of the study; East Carolina University, Greenville, North Carolina; University of North Carolina at Chapel Hill, Chapel Hill, North Carolina; University of North Carolina at Chapel Hill, Chapel Hill, North Carolina

## Abstract

A community audit is a qualitative and quantitative research technique in which researchers drive through a community to observe its physical and social attributes, primarily through windshield tours and "ground truthing." Ground truthing is a verification process that uses data gathered by direct observation to corroborate data gathered from secondary sources. Community audits have been used for epidemiologic studies and in program planning for health-promotion interventions. Few studies have detailed the methodology for conducting community audits in rural areas or the extent to which community audits can contribute to an accurate assessment of community characteristics (eg, presence of sidewalks) and nutrition and physical activity resources (eg, produce stands, parks) that may promote healthful lifestyle behaviors. The objective of this article is to describe our approach to conducting a community audit (consisting of windshield tours and ground truthing) to enumerate resources, to assess community characteristics, and to inform revisions to a community guide on nutrition and physical activity resources. We conducted an audit in 10 communities in a rural eastern North Carolina county in 2010. We also collected data from secondary sources to make comparisons with community audit data. The initial resource guide included 42 resources; the community audits identified 38 additional resources. There was moderate to high agreement between windshield tour observations and secondary data sources for several community characteristics, such as number of fast-food restaurants (67% agreement) and existence of sidewalks (100% agreement). Community audits improved the description of health-promoting community resources and the context in which people make lifestyle choices.

## Introduction

In the United States, environmental and lifestyle factors contribute to the consumption of energy-dense foods and physical inactivity, thus increasing the risk of obesity and related chronic diseases (1-5). Interventions aimed at promoting healthful lifestyle behaviors have included strategies to address environmental barriers and factors that facilitate consumption of healthful food and physical activity (6). One approach to addressing the environments in which people make lifestyle choices is to develop a guide to community nutrition and physical activity resources (7-9). Financial and administrative constraints often prohibit development of new health-promoting resources (eg, parks, trails, farmers' markets); thus, indexing and promoting the use of existing resources are good ways to encourage healthier behaviors. Additionally, establishing an accurate list of resources (ie, indexing) can help communities identify resource gaps*.*

Community resource guides are developed by using several methods, including Internet searches, interviews of community members, and direct observation (9-11). One form of direct observation is a windshield tour. Windshield tours are conducted by driving through a community of interest to directly observe and to describe its physical and social characteristics. Windshield tours have been used to inform health promotion interventions, including a teenage-pregnancy prevention program (11) and a village health worker intervention (10), and to examine how community conditions affect health behaviors (12).

Windshield tours are more robust than secondary data analysis because they provide researchers with detailed contextual information to better understand community conditions. Windshield tours are akin to "ground truthing," a verification process that uses data gathered by direct observation to corroborate data gathered from secondary sources (13). The main difference between the 2 processes is intent: a windshield tour is a qualitative observational method that examines community characteristics, whereas ground truthing is direct observation undertaken to accurately enumerate resources, usually for the purposes of validating secondary data and identifying new resources. In this study, we incorporated both methods as a combined technique, which we describe as a community audit.

In rural areas, it is often difficult to develop community resource guides because resources may not be well documented through readily available sources, and direct observation involves canvassing large geographic areas. Although a methodology for ground truthing food stores in rural areas and a general methodology for nongeographic-specific community audits have been described (13,14), no studies have detailed the methodology for conducting windshield tours and ground truthing in low-income, rural areas to identify health-promoting resources. Few studies (13,15,16) have examined the extent to which windshield tours and ground truthing corroborate resources and community context as identified through secondary data sources. One study (13) found that food-store ground truthing increased the number of identified food stores by 35.7%, whereas another study (16) found a much smaller increase (2.4%). A third study (15) found moderate to high agreement between ground truthing and food store administrative lists but less agreement for commercial physical activity venues. Another study (9) used windshield tours to inform revisions to a community resource guide initially developed from secondary data, but the study did not describe planning for the windshield tours, nor did it compare observational data with the initial guide or the secondary data.

The objective of this article is 1) to describe our approach to conducting a community audit through windshield tours and ground truthing to inform revisions to an initial community resource guide (originally developed by using secondary data sources) and to learn more about community characteristics, 2) to compare community audit findings with secondary data, and 3) to examine the advantages and disadvantages of conducting a community audit. Our goal is to provide instruction for others who wish to better understand community resources that promote healthful lifestyle behaviors.

## How to Apply Community Audit Techniques

### Develop and define objectives

The main objectives for our community audit were to verify resources listed on an initial community resource guide and to identify resources that were not listed. We collected data on the existence of the following public resources: walking trails, community parks, free or low-cost gyms (discounts to low-income members or cost of less than $20 a month), supermarkets and grocery stores, farmers' markets, and produce stands. We also collected data on the important attributes (eg, types of amenities) of each resource. In addition, we collected data on community characteristics, or *context,* which is how neighborhood-level social determinants are spatially associated with resources (17). We noted the existence of sidewalks, the number of fast-food restaurants, the proximity of convenience stores and fast-food restaurants to low-income neighborhoods, and the presence of food deserts (no supermarket or grocery store in a geographic area [18]).

### Know your audience

Identifying the target audience for a community resource guide helps inform the community audit. Our study was conducted in Pitt County, North Carolina, as part of the formative work to develop a lifestyle intervention to reduce risk for cardiovascular disease among women aged 18 to 44 years accessing reproductive health services at a Title X family planning clinic at the Pitt County Health Department. Many of these women are low-income members of racial/ethnic minority populations and thus more likely to be obese and at risk for cardiovascular disease (19-21). Our study focused on the development of a community resource guide to help link low-income women with local health-promoting resources.

We used secondary data sources, primarily the US Census and the 2009 Behavioral Risk Factor Surveillance Survey, to describe the general characteristics of our target audience. The population of Pitt County in 2009 was estimated to be 159,057 (22); approximately 20% of its residents live below the federal poverty level, and 62% of adults are overweight or obese (23). The county consists of a small urban center (population 84,986), which is home to a large public university and academic medical center, 3 small towns (population 4,615-8,586), 6 very small towns (population 112-2,240), and the surrounding rural areas. The county's area is 651.8 square miles (22). We used US Census classifications (24) to define urban and rural. *Urban* is defined as 1) core census-block groups with a population density of at least 1,000 people per square mile and 2) surrounding census-block groups with an overall density of at least 500 people per square mile. *Rural* is defined as areas outside of urban areas.

### Compile an initial resource guide by using secondary data sources

Compiling an initial resource guide provides a baseline index of what is available in a community. We developed an initial Pitt County community resource guide (hereafter referred to as "the initial guide") using Internet searches and brief interviews of community members. For example, we queried the North Carolina Farm Fresh database (www.ncfarmfresh.com) to find locations of produce stands and farmers' markets. We then asked produce stand owners about other stands and markets that were not indexed on the Farm Fresh website. In addition, we conducted broad Internet searches to find listings of parks and walking trails.

### Create field documents

Developing field documents to organize data collection ensures that variables of interest are accurately documented. We developed two 1-page field documents. The first guided resource enumeration and description. It included the list of resources from the initial guide and a checklist of items to assess during observation, including location, amenities (eg, basketball courts, pools, playgrounds), number of people using the resource, and cleanliness. The second document guided description of community characteristics, an approach previously used (11): existence of sidewalks, number of fast-food restaurants, the proximity of fast-food restaurants and convenience stores to low-income areas, and the presence of supermarkets or grocery stores.

### Conduct windshield tours and ground truthing

To organize the community audit, we divided the study area into 4 quadrants: north, south, east, and west. We assigned each of the 9 small and very small towns to a quadrant. We then mapped community resources from the initial guide within each quadrant by using Google MyMaps (25) to identify resource-dense roads and plan the best routes. A member of the research team (JM) used a vehicular global positioning satellite (GPS) system to locate each resource listed on the initial guide and visited each resource to gather information required to complete the 2 field documents. The researcher then drove the nearby main roads in an effort to identify previously unidentified resources. At the completion of each tour, the researcher spoke with community members, either at the town hall or in the planning department, to determine whether additional resources existed. (We did not arrange these interviews beforehand, and we did not use a structured interview guide.) We then visited these additional resources and gathered data.

We divided the urban center into 4 quadrants: northwest, northeast, southeast, southwest; the axes matched the major north/south and east/west roads. We developed a map for the urban center, using Google MyMaps (25). The researcher (JM) then canvassed each quadrant to enumerate resources and describe resource and community characteristics.

### Analyze findings

After the community audits are completed, the next step is to analyze findings. After all tours were completed and resources enumerated and described, we calculated the difference between the number of resources on the initial guide and the number gathered from community audits.

We then compared our observations on community characteristics with readily available secondary data sources to examine the level of agreement. Our aim was to more thoroughly understand the validity of using secondary data to examine community characteristics. We used Google Earth (26) mapping software to examine commercial and residential districts for the presence of sidewalks using both aerial and street-level views. We determined the number of fast-food restaurants through the Reference USA (27) business database. We identified restaurants on the basis of national or regional chain-name recognition and included all establishments that have designated drive-through windows or provide most of their business as take-out service or do both. We calculated fast-food restaurant density as the number of fast-food restaurants per capita in each town; the 3 towns with the greatest density were defined as having a high density of fast-food restaurants.

We examined the proximity of fast-food restaurants and convenience stores to low-income areas, because studies suggest that such food venues, which offer less healthful foods, are often more common in low-income areas (28-30). We used ArcGIS 9.3 mapping software (ESRI, Redlands, California) to assign geographic codes to fast-food restaurant and convenience store locations. We ascertained convenience store locations through Reference USA (27) according to designated North American Industrial Classification System codes (convenience stores, with and without gas pumps, code number 4451200). We conducted a road-network analysis by using ArcGIS Network Analysis and Spatial Analyst (ESRI, Redlands, California) to measure 1-mile road-network buffers from the fast-food and convenience stores. We then examined buffer overlap with low-income census-block groups (31). We defined *low-income* as a median annual household income of $36,500 or less for a family of 4, the low-income criteria established by the US Department of Housing and Urban Development for 2000 (32). We defined *close proximity* as towns in which 75% or more of the low-income census block groups were within 1 mile of fast-food restaurants or convenience stores. Research suggests that 1-mile road-network buffers indicate close proximity (30). We defined a *food desert* as a town that lacked a supermarket or grocery store. We ascertained supermarket and grocery store locations through ReferenceUSA and assigned geographic codes to locations in ArcGIS.

## Community Audit Findings

### Enumeration of resources

The initial guide included 42 resources; we identified 38 additional resources for a total of 80 resources in 6 rural and 4 urban towns (Table 1). Of the 38 additions, we identified 14 (37%) by talking with community members, and 24 (63%) by direct observation. We identified 21 community parks in addition to the 9 identified in the initial guide, the largest increase (for a total of 30 resources, 70% identified by ground truthing). No additional farmers' markets were identified. In general, the larger the town population, the greater the number of community resources. We found more additional resources in smaller towns and rural areas than we found in the urban center. We found 27 additional resources in the smaller towns and rural areas (for a total of 44 resources, 62% identified from ground truthing) and 11 additional resources in the urban center (for a total of 37 resources, 30% identified by ground truthing). All resources identified in the initial guide still existed during the study. We added the newly identified resources and made 2 corrections to the initial guide; we also added information on resource amenities.

### Community characteristics

The windshield tour observations agreed with the secondary data in the assessment of the existence of sidewalks, supermarkets, and grocery stores, and food deserts (Table 2). Our observations had only moderate agreement (67%) with secondary data in assessing fast-food restaurant density and proximity of convenience stores to low-income neighborhoods and low agreement (25%) in assessing proximity of fast-food restaurants to low-income neighborhoods.

## Considerations of Conducting Community Audits

We added a larger percentage of resources to the initial guide for the rural areas than for the urban center. We found Internet documentation for the urban center to be more complete than for the rural towns, a finding supported by recent research on the low levels of efficacy for identifying rural food venues through public directories (33). Thus, windshield tours and ground truthing are important ways to enumerate such resources in rural areas.

Emerging Internet and computer tools, such as Google MyMaps, Google Earth, and ArcGIS, allow for convenient description of community characteristics without the use of field research. There was high to moderate agreement between windshield tour observations and secondary data examination of community characteristics. Secondary data are useful to identify some, but not all, community resources. Moreover, direct observation of the community allows for collection of contextual, resource-specific data.

One strength of a community audit is community immersion, through which we were able to develop a better understanding of community and resource context, an understanding that cannot fully be obtained by examining secondary data sources alone. Informally talking with community members provided insight into commonly used resources that may be especially appealing to promote healthful lifestyle behaviors.

The resources required for our community audit were minimal. We required a single field researcher, a vehicle (walking or biking may be an option in smaller areas), two 1-page field documents, and Internet access. Although our requirements were minimal, other audits might be more costly. On-the-ground field research and secondary data analysis of communities can be resource intensive, depending on the catchment area, method, and focus of the study. Other methods, such as using secondary reference data exclusively, may avoid some of these costs but also may limit quality and completeness.

We conducted our study in 1 county in eastern North Carolina; thus, the generalizability of our study is limited. The difference in numbers of resources between the initial guide and the revised guide may be due to insufficient methods for compiling the initial guide, although we compiled the initial guide by using carefully selected search terms and interviewing community members. Descriptive assessment of community resources may have been influenced by observer bias, and analysis of community characteristics may have benefited from comparing observations from 2 field researchers. Finally, because of the grouping of households within a census-block group (600-3,000 households) (34), we may not have sufficiently represented the variation of income within census-block groups.

A community audit in a rural eastern North Carolina county yielded many additions and several alterations to an initial community resource guide. Moreover, the audit resulted in an enhanced understanding of the contextual barriers and facilitators to lifestyle change. The techniques presented in this article may serve as a model for health-promotion professionals in other rural communities.

## Figures and Tables

**Table 1.** Numbers of Community Resources Identified in the Initial Community Resource Guide (CRG) and by Subsequent Ground Truthing, Eastern North Carolina, 2010^a^

Community Resource	Towns		Total No. of Resources Identified by Each Method	Additional Resources Identified by Ground Truthing (New Resources/Total Resources)

	Rural(n = 6)	Urban(n = 4)		
**Walking trail**				
Initial CRG	5	18	23	26% (8/31)
Ground truthing	8	23	31	
**Community park**				
Initial CRG	2	7	9	70% (21/30)
Ground truthing	7	23	30	
**Free or reduced-cost gym**				
Initial CRG	0	4	4	63% (7/11)
Ground truthing	2	9	11	
**Farmers' market**				
Initial CRG	0	3	3	0% (0/3)
Ground truthing	0	3	3	
**Produce stand**				
Initial CRG	0	3	3	40% (2/5)
Ground truthing	0	5	5	
**All categories combined**				
Initial CRG	7	35	42	48% (38/80)
Ground truthing	17	63	80	

a The counts for resources identified by ground truthing represent only newly identified resources; they do not include resources in the initial CRG. All resources identified in the initial CRG still existed when the study was done.

**Table 2:** Agreement Between Information on Community Characteristics Obtained Through Windshield Tours and Secondary Data, 2010^a^

	Town Identifier^b^										No. of Towns With Characteristic	% Agreement

	A	B	C	D	E	F	G	H	I	J		
**No existing sidewalks**												
Windshield tour		x			x						2	100
Secondary data		x			x						2	
**Sidewalks in residential areas**												
Windshield tour	x			x			x		x	x	5	100
Secondary data	x			x			x		x	x	5	
**Sidewalks in commercial areas**												
Windshield tour	x		x	x		x	x	x	x	x	8	100
Secondary data	x		x	x		x	x	x	x	x	8	
**High density of fast-food restaurants**												
Windshield tour				x			x				2	67
Secondary data				x			x	x			3	
**Fast-food restaurants proximal to low-income neighborhoods**												
Windshield tour							x				1	25
Secondary data				x			x	x		x	4	
**Convenience stores proximal to low-income neighborhoods**												
Windshield tour	x	x	x				x	x			5	67
Secondary data	x		x				x	x		x	5	
**Food deserts**												
Windshield tour	x	x	x		x	x					5	100
Secondary data	x	x	x		x	x					5	

a Secondary data sources included ArcGIS software (ERSI, Redlands, California), Reference USA (27), and US Census (32).

b Towns A through F are rural towns; Towns G through J are urban towns. G is a small urban center.
